# Bis(μ_2_-pyridine-2-carboxamide oximato)bis­[(pyridine-2-carboxamide oxime)zinc] dinitrate

**DOI:** 10.1107/S1600536811034908

**Published:** 2011-08-31

**Authors:** Xiao-Hui Deng, Jing-Wen Ran

**Affiliations:** a Institute of Cash Crops, Hubei Academy of Agricultural Science, Wuhan 430064, People’s Republic of China; b Key Laboratory of Industrial Ecology and Environmental Engineering (MOE) and State Key Laboratory of Fine Chemical, School of Environmental Science and Technology, Dalian University of Technology, Dalian 116024, People’s Republic of China

## Abstract

In the title dinuclear compound, [Zn_2_(C_6_H_6_N_3_O)_2_(C_6_H_7_N_3_O)_2_](NO_3_)_2_, the Zn^II^ cation is *N*,*N*′-chelated by one pyridine-2-carboxamide oximate anion and one pyridine-2-carboxamide oxime mol­ecule, and is further bridged by an oxime O atom from the adjacent pyridine-2-carboxamide oximate anion, forming a distorted trigonal bipyramidal coordination. Two pyridine-2-carboxamide oximate anions bridge two Zn^II^ cations to form the centrosymmetric dinuclear mol­ecule. Extensive O—H⋯O, N—H⋯O and O—H⋯N hydrogen bonds are present in the crystal structure.

## Related literature

For similar metal complexes, see: Papatriantafyllopoulou *et al.* (2008 ▶); Stamatatos *et al.* (2006*a* ▶,*b* ▶). For the synthesis of the ligand, see: Bernasek (1957 ▶).
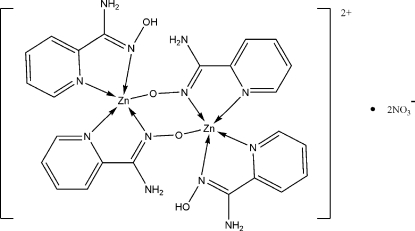


## Experimental

### 

#### Crystal data

[Zn_2_(C_6_H_6_N_3_O)_2_(C_6_H_7_N_3_O)_2_](NO_3_)_2_
*M* *_r_* = 801.33
Monoclinic, 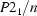

*a* = 7.4125 (14) Å
*b* = 22.201 (4) Å
*c* = 9.4225 (17) Å
β = 106.794 (2)°
*V* = 1484.5 (5) Å^3^
*Z* = 2
Mo *K*α radiation
μ = 1.70 mm^−1^
*T* = 293 K
0.51 × 0.48 × 0.39 mm

#### Data collection

Bruker SMART 1000 CCD area-detector diffractometer
Absorption correction: multi-scan (*SADABS*; Sheldrick, 1996 ▶) *T*_min_ = 0.478, *T*_max_ = 0.557
8302 measured reflections
2632 independent reflections
2297 reflections with *I* > 2σ(*I*)
*R*_int_ = 0.023

#### Refinement

*R*[*F* ^2^ > 2σ(*F* ^2^)] = 0.046
*wR*(*F* ^2^) = 0.117
*S* = 1.03
2632 reflections
209 parameters
H-atom parameters constrained
Δρ_max_ = 1.43 e Å^−3^
Δρ_min_ = −0.97 e Å^−3^

### 

Data collection: *SMART* (Bruker, 2007 ▶); cell refinement: *SAINT* (Bruker, 2007 ▶); data reduction: *SAINT*; program(s) used to solve structure: *SHELXTL* (Sheldrick, 2008 ▶); program(s) used to refine structure: *SHELXTL*; molecular graphics: *SHELXTL*; software used to prepare material for publication: *SHELXTL*.

## Supplementary Material

Crystal structure: contains datablock(s) I, global. DOI: 10.1107/S1600536811034908/xu5304sup1.cif

Structure factors: contains datablock(s) I. DOI: 10.1107/S1600536811034908/xu5304Isup2.hkl

Additional supplementary materials:  crystallographic information; 3D view; checkCIF report

## Figures and Tables

**Table 1:** Selected bond lengths (Å)

Zn1—O2	1.981 (3)
Zn1—N1	2.148 (3)
Zn1—N3	2.064 (3)
Zn1—N4	2.128 (3)
Zn1—N6	2.099 (3)

**Table 2:** Hydrogen-bond geometry (Å, °)

*D*—H⋯*A*	*D*—H	H⋯*A*	*D*⋯*A*	*D*—H⋯*A*
O1—H1⋯O2^i^	0.82	1.98	2.767 (4)	162
O1—H1⋯N6	0.82	2.43	3.018 (4)	129
N2—H2*A*⋯O2^ii^	0.86	2.32	3.108 (5)	152
N2—H2*B*⋯O5^iii^	0.86	2.35	3.180 (5)	162
N5—H5*A*⋯O4	0.86	2.18	2.900 (5)	142
N5—H5*B*⋯O5^iv^	0.86	2.18	2.985 (5)	156

Symmetry codes: (i) 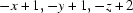; (ii) 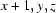; (iii) 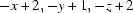; (iv) 
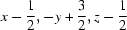
.

**Fig. 1.**
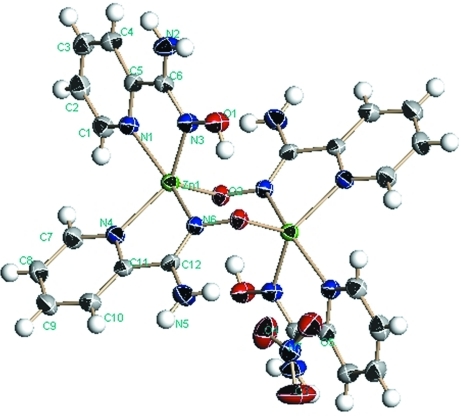
The molecular structure of (I), showing the atom-numbering scheme. Displacement ellipsoids are drawn at the 50% probability level.

**Fig. 2.**
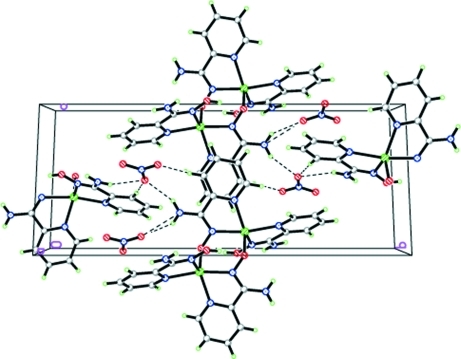
The packed diagram for the title compound, viewed down the a axis with hydrogen bonds drawn as dashed lines.

## supplementary crystallographic information

### Comment

Transition metal compounds have been of great interest for many years. They are very important in the development of coordination chemistry. As an extension of work on the structural characterization of Zn compounds, we report here the crystal structure of a new dinuclear zinc(II) compound(I) (Scheme). Compound (I) is a symmetric dinuclear ZnII complex (Fig. 1). The ZnII ion in the compound is five-coordinated by four N and an O atoms from ligands. Each of these ligands chelates one ZnII atom forming a five-membered ZnNCCN chelating ring, while its oximate oxygen atom is terminally bound to the other metal center. The Zn1—N1 and Zn1—N4 bond distances are longer than the Zn1—N3 and Zn1—N6 bond distances. The N3—Zn1—N6 angle is smaller than the N1—Zn1—N4 angle (Table 1). The molecules are stacked along the *a* axis and display N—H···O and O—H···O hydrogen-bonds interaction (Fig. 2).

### Experimental

The synthesis of pyridine-2-amidoxine was carried out according to literature (Bernasek, 1957). The title compound was synthesized by adding solid Zn(NO_3_)_2_.6H_2_O (297 mg, 1 mmol) to a solution of ligands (274 mg, 2 mmol) and NaOH (40 mg, 1 mmol) in ethanol/water (3:1, 20 ml), then the mixture was stirred for 2 h at room temperature. The solution was filtered and the filtrate was allowed to stand in air for 3 d, and yellow crystals were formed at the bottom of the vessel on slow evaporation of the solvent at room temperature. Yield: 48%. Anal. Calcd for C_24_H_26_N_14_Zn_2_O_10_: C 35.97, H 3.27, N 24.46. Found: C 35.94, H 3.29, N 23.93.

### Refinement

H atoms were included in calculated positions with C—H = 0.93 or 0.97, N—H = 086 and O—H = 0.82 Å, and refined using a riding-model with *U*_iso_(H) = 1.2U_eq_(C,N) and 1.5U_eq_(O).

### Crystal data

**Table:**

[Zn_2_(C_6_H_6_N_3_O)_2_(C_6_H_7_N_3_O)_2_](NO_3_)_2_	*F*(000) = 816
*M**_r_* = 801.33	*D*_x_ = 1.793 Mg m^−^^3^
Monoclinic, *P*2_1_/*n*	Mo *K*α radiation, λ = 0.71073 Å
Hall symbol: -P 2yn	Cell parameters from 2632 reflections
*a* = 7.4125 (14) Å	θ = 1.8–25.1°
*b* = 22.201 (4) Å	µ = 1.70 mm^−^^1^
*c* = 9.4225 (17) Å	*T* = 293 K
β = 106.794 (2)°	Block, yellow
*V* = 1484.5 (5) Å^3^	0.51 × 0.48 × 0.39 mm
*Z* = 2	

### Data collection

**Table:**

Bruker SMART 1000 CCD area-detector diffractometer	2632 independent reflections
Radiation source: fine-focus sealed tube	2297 reflections with *I* > 2σ(*I*)
graphite	*R*_int_ = 0.023
φ and ω scans	θ_max_ = 25.1°, θ_min_ = 1.8°
Absorption correction: multi-scan (*SADABS*; Sheldrick, 1996)	*h* = −8→6
*T*_min_ = 0.478, *T*_max_ = 0.557	*k* = −26→26
8302 measured reflections	*l* = −9→11

### Refinement

**Table:**

Refinement on *F*^2^	Primary atom site location: structure-invariant direct methods
Least-squares matrix: full	Secondary atom site location: difference Fourier map
*R*[*F*^2^ > 2σ(*F*^2^)] = 0.046	Hydrogen site location: inferred from neighbouring sites
*wR*(*F*^2^) = 0.117	H-atom parameters constrained
*S* = 1.03	*w* = 1/[σ^2^(*F*_o_^2^) + (0.0547*P*)^2^ + 4.2299*P*] where *P* = (*F*_o_^2^ + 2*F*_c_^2^)/3
2632 reflections	(Δ/σ)_max_ = 0.001
209 parameters	Δρ_max_ = 1.43 e Å^−^^3^
0 restraints	Δρ_min_ = −0.97 e Å^−^^3^

### Special details

**Table:**

Geometry. All e.s.d.'s (except the e.s.d. in the dihedral angle between two l.s. planes) are estimated using the full covariance matrix. The cell e.s.d.'s are taken into account individually in the estimation of e.s.d.'s in distances, angles and torsion angles; correlations between e.s.d.'s in cell parameters are only used when they are defined by crystal symmetry. An approximate (isotropic) treatment of cell e.s.d.'s is used for estimating e.s.d.'s involving l.s. planes.
Refinement. Refinement of *F*^2^ against ALL reflections. The weighted *R*-factor *wR* and goodness of fit *S* are based on *F*^2^, conventional *R*-factors *R* are based on *F*, with *F* set to zero for negative *F*^2^. The threshold expression of *F*^2^ > σ(*F*^2^) is used only for calculating *R*-factors(gt) *etc*. and is not relevant to the choice of reflections for refinement. *R*-factors based on *F*^2^ are statistically about twice as large as those based on *F*, and *R*- factors based on ALL data will be even larger.

### Fractional atomic coordinates and isotropic or equivalent isotropic displacement parameters (Å^2^)

**Table:**

	*x*	*y*	*z*	*U*_iso_*/*U*_eq_	
Zn1	0.46944 (6)	0.437379 (19)	0.86499 (5)	0.02829 (17)	
O2	0.3307 (4)	0.44030 (11)	1.0158 (3)	0.0305 (6)	
N6	0.5478 (4)	0.52822 (14)	0.8642 (3)	0.0279 (7)	
O1	0.8933 (4)	0.45773 (13)	1.0281 (4)	0.0412 (7)	
H1	0.8498	0.4919	1.0208	0.062*	
C12	0.4742 (5)	0.55977 (16)	0.7481 (4)	0.0290 (8)	
N3	0.7493 (5)	0.41671 (15)	0.9631 (4)	0.0326 (7)	
C11	0.3546 (5)	0.52544 (17)	0.6194 (4)	0.0302 (8)	
N1	0.4877 (5)	0.34274 (15)	0.8238 (4)	0.0321 (7)	
N4	0.3239 (5)	0.46677 (15)	0.6470 (4)	0.0311 (7)	
N5	0.5021 (6)	0.61900 (15)	0.7361 (4)	0.0465 (10)	
H5A	0.5739	0.6385	0.8099	0.056*	
H5B	0.4482	0.6375	0.6547	0.056*	
N7	0.6922 (6)	0.77875 (19)	0.9401 (5)	0.0590 (5)	
N2	0.9964 (5)	0.35112 (17)	0.9759 (4)	0.0429 (9)	
H2A	1.0797	0.3778	1.0161	0.051*	
H2B	1.0309	0.3156	0.9583	0.051*	
C5	0.6647 (5)	0.32001 (17)	0.8733 (4)	0.0298 (8)	
C8	0.1400 (7)	0.4564 (2)	0.3942 (5)	0.0431 (11)	
H8	0.0665	0.4321	0.3194	0.052*	
C10	0.2807 (6)	0.55061 (19)	0.4812 (5)	0.0383 (10)	
H10	0.3038	0.5908	0.4647	0.046*	
C6	0.8138 (5)	0.36488 (17)	0.9409 (4)	0.0298 (8)	
O5	0.8612 (5)	0.78443 (16)	1.0109 (4)	0.0590 (5)	
O3	0.5941 (5)	0.82406 (15)	0.9079 (4)	0.0590 (5)	
O4	0.6244 (5)	0.72889 (15)	0.9025 (4)	0.0590 (5)	
C7	0.2194 (7)	0.43374 (19)	0.5347 (5)	0.0406 (10)	
H7	0.1992	0.3934	0.5520	0.049*	
C4	0.7013 (7)	0.25955 (19)	0.8636 (5)	0.0402 (10)	
H4	0.8237	0.2449	0.8987	0.048*	
C3	0.5540 (7)	0.22116 (19)	0.8011 (5)	0.0462 (11)	
H3	0.5762	0.1803	0.7922	0.055*	
C9	0.1715 (6)	0.5154 (2)	0.3668 (5)	0.0431 (11)	
H9	0.1203	0.5317	0.2729	0.052*	
C1	0.3466 (6)	0.30447 (19)	0.7682 (5)	0.0380 (10)	
H1A	0.2242	0.3194	0.7389	0.046*	
C2	0.3739 (7)	0.2436 (2)	0.7522 (5)	0.0428 (11)	
H2	0.2727	0.2184	0.7092	0.051*	

### Atomic displacement parameters (Å^2^)

**Table:**

	*U*^11^	*U*^22^	*U*^33^	*U*^12^	*U*^13^	*U*^23^
Zn1	0.0276 (3)	0.0250 (3)	0.0300 (3)	0.00004 (17)	0.00468 (19)	−0.00044 (17)
O2	0.0287 (14)	0.0296 (14)	0.0315 (14)	−0.0055 (11)	0.0062 (12)	−0.0041 (11)
N6	0.0281 (16)	0.0260 (16)	0.0276 (17)	−0.0017 (13)	0.0049 (13)	−0.0021 (13)
O1	0.0293 (15)	0.0309 (15)	0.0560 (19)	−0.0054 (12)	0.0003 (14)	−0.0053 (14)
C12	0.030 (2)	0.0242 (18)	0.033 (2)	0.0029 (15)	0.0079 (17)	−0.0004 (15)
N3	0.0253 (17)	0.0295 (17)	0.0394 (19)	−0.0038 (14)	0.0039 (14)	−0.0009 (14)
C11	0.029 (2)	0.0298 (19)	0.032 (2)	0.0051 (16)	0.0094 (16)	0.0001 (16)
N1	0.0302 (17)	0.0287 (17)	0.0348 (18)	−0.0006 (14)	0.0053 (14)	−0.0003 (14)
N4	0.0341 (18)	0.0279 (16)	0.0286 (17)	0.0006 (14)	0.0048 (14)	−0.0003 (13)
N5	0.061 (3)	0.0255 (18)	0.042 (2)	−0.0020 (17)	−0.0027 (19)	0.0038 (15)
N7	0.0512 (11)	0.0453 (9)	0.0727 (13)	−0.0021 (8)	0.0056 (9)	−0.0090 (9)
N2	0.0279 (18)	0.0374 (19)	0.059 (2)	0.0032 (15)	0.0050 (17)	−0.0044 (17)
C5	0.033 (2)	0.0289 (19)	0.029 (2)	0.0016 (16)	0.0098 (16)	0.0010 (16)
C8	0.045 (3)	0.046 (3)	0.033 (2)	−0.002 (2)	0.0034 (19)	−0.0070 (19)
C10	0.043 (2)	0.035 (2)	0.037 (2)	0.0052 (18)	0.0099 (19)	0.0056 (18)
C6	0.029 (2)	0.030 (2)	0.029 (2)	0.0030 (16)	0.0057 (16)	0.0052 (16)
O5	0.0512 (11)	0.0453 (9)	0.0727 (13)	−0.0021 (8)	0.0056 (9)	−0.0090 (9)
O3	0.0512 (11)	0.0453 (9)	0.0727 (13)	−0.0021 (8)	0.0056 (9)	−0.0090 (9)
O4	0.0512 (11)	0.0453 (9)	0.0727 (13)	−0.0021 (8)	0.0056 (9)	−0.0090 (9)
C7	0.046 (3)	0.032 (2)	0.037 (2)	−0.0053 (18)	0.002 (2)	−0.0044 (18)
C4	0.044 (2)	0.033 (2)	0.043 (3)	0.0053 (18)	0.011 (2)	0.0009 (18)
C3	0.061 (3)	0.028 (2)	0.048 (3)	0.000 (2)	0.013 (2)	−0.0053 (19)
C9	0.043 (3)	0.051 (3)	0.032 (2)	0.006 (2)	0.0056 (19)	0.0050 (19)
C1	0.035 (2)	0.038 (2)	0.038 (2)	−0.0052 (18)	0.0059 (18)	0.0014 (18)
C2	0.050 (3)	0.039 (2)	0.038 (2)	−0.015 (2)	0.010 (2)	−0.0048 (19)

### Geometric parameters (Å, °)

**Table:**

Zn1—O2	1.981 (3)	N7—O4	1.225 (5)
Zn1—N1	2.148 (3)	N7—O5	1.244 (5)
Zn1—N3	2.064 (3)	N2—C6	1.333 (5)
Zn1—N4	2.128 (3)	N2—H2A	0.8600
Zn1—N6	2.099 (3)	N2—H2B	0.8600
O2—N6^i^	1.411 (4)	C5—C4	1.378 (6)
N6—C12	1.281 (5)	C5—C6	1.487 (5)
N6—O2^i^	1.411 (4)	C8—C9	1.368 (7)
O1—N3	1.402 (4)	C8—C7	1.379 (6)
O1—H1	0.8200	C8—H8	0.9300
C12—N5	1.341 (5)	C10—C9	1.386 (6)
C12—C11	1.489 (5)	C10—H10	0.9300
N3—C6	1.286 (5)	C7—H7	0.9300
C11—N4	1.361 (5)	C4—C3	1.376 (6)
C11—C10	1.377 (6)	C4—H4	0.9300
N1—C1	1.332 (5)	C3—C2	1.374 (7)
N1—C5	1.357 (5)	C3—H3	0.9300
N4—C7	1.336 (5)	C9—H9	0.9300
N5—H5A	0.8600	C1—C2	1.381 (6)
N5—H5B	0.8600	C1—H1A	0.9300
N7—O3	1.227 (5)	C2—H2	0.9300
			
O2—Zn1—N3	110.42 (13)	O4—N7—O5	120.8 (4)
O2—Zn1—N6	99.93 (12)	C6—N2—H2A	120.0
N3—Zn1—N6	88.39 (13)	C6—N2—H2B	120.0
O2—Zn1—N4	117.30 (12)	H2A—N2—H2B	120.0
N3—Zn1—N4	131.62 (14)	N1—C5—C4	121.9 (4)
N6—Zn1—N4	76.45 (12)	N1—C5—C6	115.0 (3)
O2—Zn1—N1	103.55 (12)	C4—C5—C6	123.0 (4)
N3—Zn1—N1	75.87 (13)	C9—C8—C7	118.8 (4)
N6—Zn1—N1	155.12 (13)	C9—C8—H8	120.6
N4—Zn1—N1	99.58 (13)	C7—C8—H8	120.6
N6^i^—O2—Zn1	104.3 (2)	C9—C10—C11	119.3 (4)
C12—N6—O2^i^	115.4 (3)	C9—C10—H10	120.3
C12—N6—Zn1	118.6 (3)	C11—C10—H10	120.3
O2^i^—N6—Zn1	125.9 (2)	N3—C6—N2	124.3 (4)
N3—O1—H1	109.5	N3—C6—C5	113.8 (3)
N6—C12—N5	124.8 (4)	N2—C6—C5	121.9 (4)
N6—C12—C11	115.0 (3)	N4—C7—C8	123.3 (4)
N5—C12—C11	120.2 (4)	N4—C7—H7	118.3
C6—N3—O1	112.4 (3)	C8—C7—H7	118.3
C6—N3—Zn1	119.9 (3)	C5—C4—C3	119.0 (4)
O1—N3—Zn1	126.2 (2)	C5—C4—H4	120.5
N4—C11—C10	121.8 (4)	C3—C4—H4	120.5
N4—C11—C12	115.3 (3)	C2—C3—C4	119.5 (4)
C10—C11—C12	122.9 (4)	C2—C3—H3	120.2
C1—N1—C5	118.0 (3)	C4—C3—H3	120.2
C1—N1—Zn1	127.7 (3)	C8—C9—C10	119.1 (4)
C5—N1—Zn1	114.0 (2)	C8—C9—H9	120.4
C7—N4—C11	117.7 (3)	C10—C9—H9	120.4
C7—N4—Zn1	127.8 (3)	N1—C1—C2	123.0 (4)
C11—N4—Zn1	114.3 (2)	N1—C1—H1A	118.5
C12—N5—H5A	120.0	C2—C1—H1A	118.5
C12—N5—H5B	120.0	C3—C2—C1	118.5 (4)
H5A—N5—H5B	120.0	C3—C2—H2	120.8
O3—N7—O4	120.3 (4)	C1—C2—H2	120.8
O3—N7—O5	118.9 (4)		
			
N3—Zn1—O2—N6^i^	43.2 (2)	C12—C11—N4—C7	−179.2 (4)
N6—Zn1—O2—N6^i^	−48.8 (2)	C10—C11—N4—Zn1	175.9 (3)
N4—Zn1—O2—N6^i^	−128.6 (2)	C12—C11—N4—Zn1	−3.6 (4)
N1—Zn1—O2—N6^i^	123.0 (2)	O2—Zn1—N4—C7	−90.2 (4)
O2—Zn1—N6—C12	−112.5 (3)	N3—Zn1—N4—C7	100.1 (4)
N3—Zn1—N6—C12	137.0 (3)	N6—Zn1—N4—C7	175.4 (4)
N4—Zn1—N6—C12	3.4 (3)	N1—Zn1—N4—C7	20.5 (4)
N1—Zn1—N6—C12	86.9 (4)	O2—Zn1—N4—C11	94.8 (3)
O2—Zn1—N6—O2^i^	64.1 (3)	N3—Zn1—N4—C11	−74.9 (3)
N3—Zn1—N6—O2^i^	−46.3 (3)	N6—Zn1—N4—C11	0.4 (3)
N4—Zn1—N6—O2^i^	−180.0 (3)	N1—Zn1—N4—C11	−154.5 (3)
N1—Zn1—N6—O2^i^	−96.5 (4)	C1—N1—C5—C4	1.8 (6)
O2^i^—N6—C12—N5	−1.5 (6)	Zn1—N1—C5—C4	176.4 (3)
Zn1—N6—C12—N5	175.5 (3)	C1—N1—C5—C6	−177.0 (4)
O2^i^—N6—C12—C11	176.8 (3)	Zn1—N1—C5—C6	−2.4 (4)
Zn1—N6—C12—C11	−6.3 (5)	N4—C11—C10—C9	0.2 (7)
O2—Zn1—N3—C6	109.2 (3)	C12—C11—C10—C9	179.6 (4)
N6—Zn1—N3—C6	−150.8 (3)	O1—N3—C6—N2	0.3 (6)
N4—Zn1—N3—C6	−80.6 (3)	Zn1—N3—C6—N2	167.2 (3)
N1—Zn1—N3—C6	9.7 (3)	O1—N3—C6—C5	179.5 (3)
O2—Zn1—N3—O1	−86.0 (3)	Zn1—N3—C6—C5	−13.7 (5)
N6—Zn1—N3—O1	14.0 (3)	N1—C5—C6—N3	10.2 (5)
N4—Zn1—N3—O1	84.3 (3)	C4—C5—C6—N3	−168.6 (4)
N1—Zn1—N3—O1	174.6 (3)	N1—C5—C6—N2	−170.7 (4)
N6—C12—C11—N4	6.5 (5)	C4—C5—C6—N2	10.6 (6)
N5—C12—C11—N4	−175.1 (4)	C11—N4—C7—C8	−1.0 (7)
N6—C12—C11—C10	−173.0 (4)	Zn1—N4—C7—C8	−175.8 (4)
N5—C12—C11—C10	5.4 (6)	C9—C8—C7—N4	1.1 (8)
O2—Zn1—N1—C1	62.8 (4)	N1—C5—C4—C3	0.4 (7)
N3—Zn1—N1—C1	170.8 (4)	C6—C5—C4—C3	179.0 (4)
N6—Zn1—N1—C1	−136.9 (4)	C5—C4—C3—C2	−1.1 (7)
N4—Zn1—N1—C1	−58.5 (4)	C7—C8—C9—C10	−0.5 (7)
O2—Zn1—N1—C5	−111.2 (3)	C11—C10—C9—C8	−0.1 (7)
N3—Zn1—N1—C5	−3.2 (3)	C5—N1—C1—C2	−3.3 (6)
N6—Zn1—N1—C5	49.1 (5)	Zn1—N1—C1—C2	−177.1 (3)
N4—Zn1—N1—C5	127.5 (3)	C4—C3—C2—C1	−0.4 (7)
C10—C11—N4—C7	0.3 (6)	N1—C1—C2—C3	2.6 (7)

Symmetry codes: (i) −*x*+1, −*y*+1, −*z*+2.

### Hydrogen-bond geometry (Å, °)

**Table:**

*D*—H···*A*	*D*—H	H···*A*	*D*···*A*	*D*—H···*A*
O1—H1···O2^i^	0.82	1.98	2.767 (4)	162.
O1—H1···N6	0.82	2.43	3.018 (4)	129.
N2—H2A···O2^ii^	0.86	2.32	3.108 (5)	152.
N2—H2B···O5^iii^	0.86	2.35	3.180 (5)	162.
N5—H5A···O4	0.86	2.18	2.900 (5)	142.
N5—H5B···O5^iv^	0.86	2.18	2.985 (5)	156.

Symmetry codes: (i) −*x*+1, −*y*+1, −*z*+2; (ii) *x*+1, *y*, *z*; (iii) −*x*+2, −*y*+1, −*z*+2; (iv) *x*−1/2, −*y*+3/2, *z*−1/2.
